# HPTLC-*aptastaining* – Innovative protein detection system for high-performance thin-layer chromatography

**DOI:** 10.1038/srep26665

**Published:** 2016-05-25

**Authors:** Lena Morschheuser, Hauke Wessels, Christina Pille, Judith Fischer, Tim Hünniger, Markus Fischer, Angelika Paschke-Kratzin, Sascha Rohn

**Affiliations:** 1 University of Hamburg, Hamburg School of Food Science, Institute of Food Chemistry, Grindelallee 117, D-20146 Hamburg.

## Abstract

Protein analysis using high-performance thin-layer chromatography (HPTLC) is not commonly used but can complement traditional electrophoretic and mass spectrometric approaches in a unique way. Due to various detection protocols and possibilities for hyphenation, HPTLC protein analysis is a promising alternative for e.g., investigating posttranslational modifications. This study exemplarily focused on the investigation of lysozyme, an enzyme which is occurring in eggs and technologically added to foods and beverages such as wine. The detection of lysozyme is mandatory, as it might trigger allergenic reactions in sensitive individuals. To underline the advantages of HPTLC in protein analysis, the development of innovative, highly specific staining protocols leads to improved sensitivity for protein detection on HPTLC plates in comparison to universal protein derivatization reagents. This study aimed at developing a detection methodology for HPTLC separated proteins using aptamers. Due to their affinity and specificity towards a wide range of targets, an aptamer based staining procedure on HPTLC (HPTLC-*aptastaining*) will enable manifold analytical possibilities. Besides the proof of its applicability for the very first time, (i) aptamer-based staining of proteins is applicable on different stationary phase materials and (ii) furthermore, it can be used as an approach for a semi-quantitative estimation of protein concentrations.

The analysis of non-denatured proteins, especially proteoforms, is still challenging due to their multitude of structural and physicochemical characteristics. To overcome some of the problems while using LC-MS or electrophoresis, high-performance thin-layer chromatography (HPTLC) represents an interesting complementary technique^1^,^2^,^3^,^4^. In contrast to the dominantly used electrophoretic approaches, more degrees of freedom with regard to separation and detection are available.

Traditionally, detecting and quantifying proteins on HPTLC plates can be performed with fluorescamine or ninhydrin as staining reagents^5^,^6^. A more elegant approach would focus on a highly specific detection of single proteins using specific antibodies. Meisen *et al*.^7^. showed an antibody based detection on HPTLC plates for analyzing Shiga toxin binding glycosphingolipids^7^. Furthermore, we already developed an immunostaining protocol for the investigation of phosphopeptides using commercially available antibodies in a HPTLC assay^8^. In this case, the detection was carried out indirectly using a fluorescent-labelled secondary antibody.

Despite the high affinity and specificity of antibodies, the number of antigens is limited due to a certain degree of toxicity of the immunogen, by the similarity to host proteins, and by the molecular weight. The mandatory need of animals is one of the main disadvantages, because the production procedure of antibodies is rather cruel for the animal and therefore getting more and more restricted in many countries, e.g., by the European legislation (directive 2010/63/EU)^9^.

In this context, aptamers show promising characteristics to replace or rather complement the immunological analysis of proteins. Aptamers are short single-stranded RNA or DNA oligonucleotides of up to 100 nucleobases^10^,^11^. Intramolecular base pairing resulting from hydrogen bonding leads to a specific arrangement of loops and pockets resulting in defined three-dimensional structures. Hence, a high affinity and specificity for a broad range of targets (ions, small molecules, proteins, spores, or cells) was observed and published previously^12^,^13^. So far, aptamers are successfully applied as specific tools in the enrichment and analysis of certain target molecules, ranging from ions to whole cells^14^. In contrast to the production of antibodies, aptamer manufacturing is performed *in vitro* base-per-base enabling notably more precise modifications and therefore a broader range of possible structural designs^15^. In the context of chromatographic and electrophoretic separation, aptamers have been successfully applied as stationary phases in affinity chromatography^16^, and as a potential detection reagent following western blotting^17^. However, the aptamers used in the latter study only exhibited specificity against an inserted tag, but not against the protein itself^17^. Due to these properties aptamers are also a well-suited detection reagent in chromatographic applications such as HPTLC, for instance. As a result, a combination of HPTLC and aptamers, two powerful analytical tools, would lead to a promising methodology for analysing several substance classes.

The aim of this study was to develop a combination of HPTLC and a novel aptamer-based protocol (HPTLC-*aptastaining,* HPTLC-AS) for the detection of proteins following their chromatographic separation. Lysozyme, an enzyme with broad relevance in food technology, was chosen as a model protein. For example, it is used as a fining agent in white wine production for delaying or preventing malolactic fermentation^18^. Because of its allergenicity, the use of lysozyme as an additive has to be declared according to commission regulation (EU) No 1266/2010. Therefore, highly sensitive methods for lysozyme determination are mandatory for consumer protection^19^. In this study, a hen’s egg-white preparation and a lysozyme-containing fining agent were separated on different stationary phase materials such as silica gel and reversed phases followed by the detection using fluorescently labelled highly specific aptamers^20^.

## Results

### Protein chromatography

Initially, a chromatographic separation of the chosen model proteins was performed. With regard to hydropathy (GRAVY score), molecular weight, and isoelectric point, β-lactoglobulin and insulin were chosen in addition to lysozyme (according to Biller *et al*.)^4^. By evaluating different model proteins regarding their (physicochemical) properties, it should be emphasized that the chromatographic conditions can be applied to a broad range of analytical questions. To survey the various application possibilities of an aptamer-based staining procedure on HPTLC plates, the following stationary phase materials were tested: silica gel, cellulose as well as different reversed-phase (RP). Since the aptamer-based detection was supposed to be performed directly on HPTLC-plates, stationary phase materials need to be stable towards an incubation in aqueous solutions. On this account, water-wettable (W) RP-18 layers (RP-18W) were applied *inter alia*. The optimized solvent systems were partly based on manufacturer information or developed according to Biller et al. 2015, respectively^4^.

**Figure 1:**
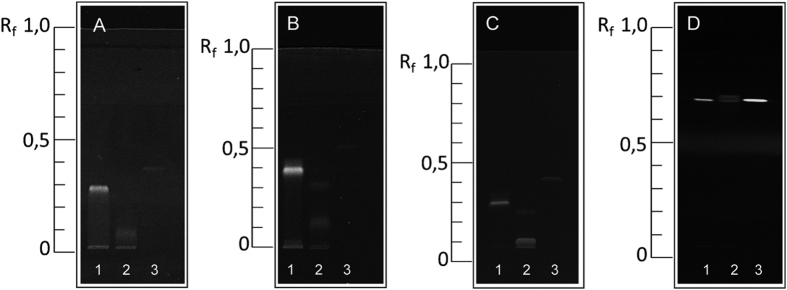
Separation of model proteins depending on different stationary phase materials: (**A**) RP-8, (**B**) RP-18, (**C**) RP-18W, and (**D**) silica gel. Derivatization was performed with fluorescamine, detected at λ = 355 nm. Legend: (1): lysozyme, (2): β-lactoglobulin, (3): insulin.

Figure 1 shows the successful separation of the model proteins on different stationary phase materials. All proteins exhibited an R_f_ value between 0.10 and 0.69. The model protein β-lactoglobulin showed two bands when using both silica gel and RP-18 plates. Presumably, this might be caused by the separation of a dimer and monomer structure of this protein^21^. However, a chromatographic separation on cellulose material was not accomplished for any of the investigated proteins. Nevertheless, to show the feasibility of the presented methodology in principle, proteins were spotted on cellulose plates and stained with the *aptastaining*-protocol without initial chromatographic separation.

### Aptamer selection

Following the successful evaluation of chromatographic conditions for silica gel and RP phases, aptamers with an affinity to lysozyme were used^15^. Initially, a so-called SELEX (*systematic evolution of ligands by exponential enrichment*) protocol has to be performed^22^. After this turn-based procedure, aptamer sequences with the desired binding properties can be identified^23^,^24^. Aptamers with an affinity towards lysozyme were identified previously and exhibited promising dissociation constants within a low nanomolar range^20^. As lysozyme is applied in white wine processing, the authors used a white wine mimicking buffer as selection buffer. Due to the influence on the secondary structure of the aptamer and thus, on its affinity, this buffer was also used in the development of the HPTLC-*aptastaining* procedure as incubation medium. To enable fluorescence detection, the aptamers were purchased after the selection process commercially with a 5′-fluorescent label (ATTO 550).

### The aptamer-based staining procedure

**Figure 2:**
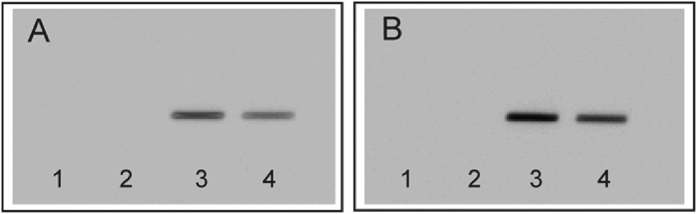
Surface enhanced analysis on two different stationary phase materials without development: (**A**) RP 18W and (**B**) silica gel. Visualization was performed after incubation in white wine buffer to proof the principle of detection (λ = 550 nm). Legend: (1): unstained protein lysozyme, (2): aptamer LysApt5, (3): fluorescently labelled aptamer 5′-ATTO 550-LysApt5, and (4) the applied fluorescence dye ATTO 550.

Following the choice of the appropriate aptamer(s) and the successful evaluation of the chromatographic conditions for the model proteins on silica gel and RP phases, the aptamer-based staining procedure was evaluated. Primarily, the suitability of the chosen documentation regarding the stability of the aptamer, the protein(s) and the fluorophore under white wine mimicking conditions was in focus. The white wine mimicking buffer was used during the SELEX process of the inserted aptamers, so the conditions are crucial for the aptamer-protein interaction. The target protein as well as the aptamer (LysApt5)^20^, the fluorescently labelled aptamer (5′-ATTO 550-LysApt5), and the fluorescence dye were applied on silica gel and RP-18W plates. The undeveloped plates were dipped in incubation medium to proof the stability of the stationary phases as well as the documentation in general. Figure 2 shows the corresponding surface enhanced analysis. Signals occurred for the fluorescently labelled aptamer (Fig. 2A,B, lane 3) and its fluorophore (Fig. 2A,B, lane 4), exclusively. So, neither the protein (Fig. 2A,B, lane 1), nor the pure aptamer (Fig. 2A,B, lane 2) appeared. With regard to the stability of the stationary phase material, no damage was observed.

Since lysozyme was successfully detected with the developed aptamer-staining protocol, (model) proteins were separated chromatographically on HPTLC-plates and incubated consecutively with fluorescently labelled aptamers dissolved in white wine buffer. The procedure was complemented by a preceding blocking step. Without suppressing non-specific binding, no signals are detectable at all (data not shown). Different blocking agents such as BSA, milk powder, and Tween^®^20, as well as various procedures were tested. Blocking with proteins led to a major background signal after the *aptastaining*. The final protocol using Tween^®^20 is described in the experimental section.

**Figure 3:**
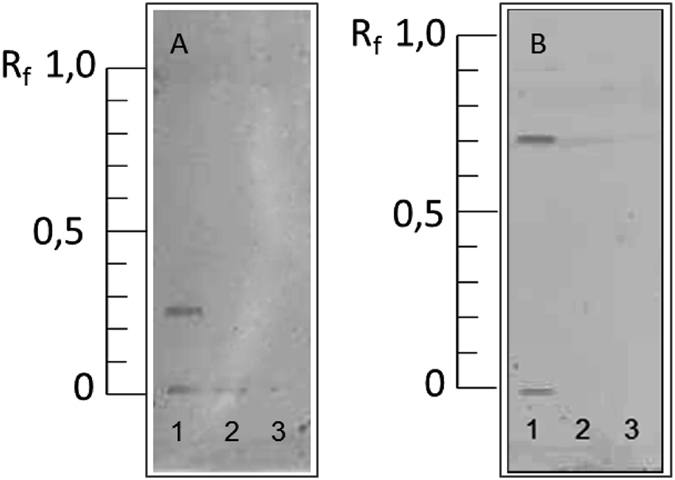
HPTLC-*aptastaining* (HPTLC-AS) of model proteins on (**A**) RP-18W and (**B**) silica gel. *Aptastaining* was performed using the aptamer LysApt5 (λ = 550 nm). Legend: (1): lysozyme, (2): β-lactoglobulin, (3): insulin.

At the beginning of this work, signals occurred for all model proteins resulting from unspecific binding of the aptamers. To minimize these interferences during the staining procedure, the aptamer concentration and the duration of incubation were reduced. Additionally, washing steps of different durations were applied to eliminate unspecifically bound aptamer residues. Applying the evolved procedure, lysozyme was successfully detected on all plate materials. Figure 3 shows exemplarily HPTLC-AS chromatograms on RP-18W and silica gel, which turned out to be most suitable for post chromatographic aptamer-based staining. HPTLC-AS on RP-8 and regular RP-18 phases led to impaired band sharpness, while HPTLC-AS on cellulose was only performed without separating the proteins (data not shown). Due to the lack of a chromatographic separation, cellulose material is not applicable in this study but is compatible with an aptamer based staining in general and bears the possibility to be used for other analytes. Between water-wettable and regular RP phases little differences were observable: The RP-18W phase showed a more rapid moistening than the RP-18, but both stationary materials demonstrated the same grade of stability. With regard to sensitivity, HPTLC-AS on RP-phases required a lower concentration of aptamers than silica gel material, presumably caused by a lower diffusion into the stationary phase material resulting from the apolar structure. In comparison to the lysozyme stained with fluorescamine (Fig. 1), HPTLC-AS (Fig. 3) showed bands with declined band sharpness, so very slightly separated analytes might smear. This is caused by horizontal diffusion effects as the plates are incubated for about 12 min in total. Nevertheless, the selective detection of single proteins was achieved.

Similarly to antibodies, also aptamers might show cross-reactivity towards other proteins, especially when applied at high concentrations. In order to analyze the cross-reactivity of the aptamer used (LysApt5), the signal intensities of other model proteins after HPTLC-AS were considered. While lysozyme was detected by *aptastaining* on RP-18W and silica gel phases exclusively; insulin and β-lactoglobulin did not show any signals (Fig. 3). However, when using RP-8 and regular RP-18 phases, insulin and β-lactoglobulin showed little cross-reactivity (Supplementary Information Figure S1). This might be probably caused by electrostatic interactions between positive charged protein side chains and the negative charged phosphate backbone of the aptamer in combination with a more apolar stationary phase material. Taking SPR data into account, the aptamer with an affinity to lysozyme showed almost no affinity towards β-lactoglobulin and insulin, too. This led to the conclusion that the cross-reactivity against other proteins is not an effect of the HPTLC-AS, but a general property of the aptamer(s). As the specific bands are separated from non-specific ones and the comparison with standards/controls allows the identification of the specific band, it is not problematic in this case. However, this study focused primarily on pointing out the applicability of aptamers as a detection system in HPTLC analysis. In order to avoid aptamer binding to other proteins or sample components, precautions can be already taken in the SELEX process by inserting a counter selection^25^.

As described in the literature, various pre-treatments of the HPTLC-plate can be applied to improve overlay (detection) procedures^26^,^27^,^28^. In order to induce the best success in detecting specific proteins using HPTLC-AS, various procedures were investigated. Due to the rapid process of HPTLC-AS, neither a fixation of the stationary phase material was necessary^26^ nor a neutralization of the HPTLC-plate^27^ led to an improved result. A renaturation of the proteins after chromatography, as described for an antibody-based assay detecting gangliosides^28^, led to band-broadening and an overall less satisfying result, consequently. So, in contrast to other staining protocols, the HPTLC-AS procedure (see Supplementary Information for an overview scheme, Figure S2) is less elaborate and succeeds without any preliminary proceedings (analysis time for AS on RP-18W approx. 2 hours).

To ensure the reliability of HPTLC-AS, the method was repeated using another aptamer with affinity to lysozyme (Table 1, LysApt4) which showed comparable dissociation constants: no obvious differences were observed. In order to proof HPTLC-AS detecting different proteins than lysozyme, aptamers with affinity towards caseins were selected and successfully used to detect caseins via HPTLC-AS in different samples (data not shown).

### HPTLC-AS for identifying lysozyme or egg-white powder in food and beverages

**Figure 4:**
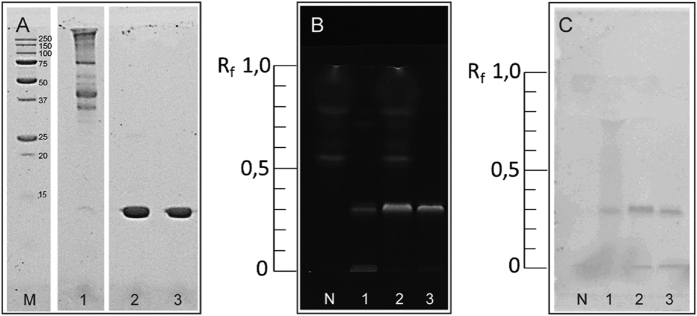
Traditionally applied SDS-PAGE of lysozyme containing samples compared to unselectively stained HPTLC and HPTLC-AS on RP-18W layers. Figure 4A shows the SDS-PAGE stained with a protein specific reagent (colloidal coomassie detection, visualized at white light). Figure 4B presents an unselectively stained HPTLC separation (fluorescamine staining solution, λ = 366 nm). The corresponding selective detection via HPTLC-AS is represented in Fig. 4C, stained with the aptamer LysApt5 (λ = 550 nm). Legend: (M): molecular weight marker (kDa), (1): powdered egg-white extracted with white wine mimicking buffer, (2): white wine spiked with lysozyme containing fining agent, (3): fining agent extracted with white wine mimicking buffer, (N): negative control (untreated white wine).

To prove the applicability of the newly developed HPTLC-AS method, different protein mixtures and commercial preparations were separated and *aptastained*. Besides an egg-white powder, a lysozyme-containing fining agent from the wine industry was analyzed. Traditionally, fining agents are proteins or further biomacromolecules that help improving percolation during the winemaking process, as a turbidity is unwanted. Analyses were conducted on RP-18, RP-18W, and silica gel phases. Primarily, samples were investigated by traditionally performed SDS-PAGE to confirm the presence of lysozyme. Figure 4 illustrates the SDS-PAGE results (Fig. 4A), a thin-layer chromatogram on a RP-18W phase stained with fluorescamine (Fig. 4B), and a corresponding plate using *aptastaining* (Fig. 4C). As expected, the egg-white powder preparation contained lysozyme and further egg-white proteins such as ovalbumin (Fig. 4A, lane 1). The lysozyme band (14 kDa) is hardly visible with Coomassie staining but visible using *aptastaining*. When comparing SDS-PAGE (Fig. 4A, lane 1) and HPTLC separation (Fig. 4B,C, lane 1), only lysozyme shows a separation (R_f_ 0.29). As reported by Biller *et al*., thin-layer chromatographic separation of ovalbumin cannot be completely accomplished under these conditions^4^. However, a clean-up effect of the sample was achieved resulting in a distinct lysozyme band.

Recent publications show the interaction between plant phenolic compounds, present in high concentrations in (white) wine, and proteins which are the main part of protein-based fining agents^29^,^30^,^31^,^32^. Exemplarily, the analysis of lysozyme in presence of phenolic compounds using RP-18W plates is shown in Fig. 4 (lane 2&3). Due to the superior separation of phenolic compounds and lysozyme on RP-18W plates (in comparison to silica plates), this chromatographic system seems to be appropriate in related research fields.

To prove the feasibility of HPTLC-AS for detecting proteins besides secondary plant metabolites, commercially available white wine was spiked with a lysozyme-containing fining agent and analyzed by HPTLC with both detection possibilities – fluorescamine and *aptastaining* (Fig. 4B,C, lane 2). Of note, neither the phenolic compounds hamper the detection of lysozyme nor are these compounds detected by the aptamer. Compared to traditional SDS-PAGE and the specific detection of single proteins, an electrophoretic separation is not appropriate for analyzing secondary plant metabolites as it is by HPTLC (Fig. 4B, lane 3). HPTLC represents an outstanding method for following protein interactions with secondary plant metabolites and the corresponding formation of posttranslational modifications^33^.

Taking the results into account, one can conclude that HPTLC-AS works in model systems as well as for the analysis of food. Neither the presence of other proteins (ovalbumin in egg-white) nor substances such as secondary plant metabolites or ethanol (white wine) hamper the analysis. Rather, HPTLC-AS can be an excellent tool for analyzing reactions between different substance classes.

Besides the successful proof of protein detection and identification by using lysozyme specific aptamers, even the feasibility of a semi-quantitative estimation was tested. For this purpose, various statistical parameters were investigated using HPTLC-AS on silica gel 60 layers. Silica gel layers were chosen as they are applicable in analyzing a broad range of analytes (including the proteins as presented herein).

Initially, the concentrations with a linear correlation to the concentration of lysozyme were investigated. Briefly, the method shows a linearity between 0.1 μg and 1 μg lysozyme, confirmed by a residual analysis of all calibration standards. Additionally, linearity was confirmed by means of Mandels fitting test. Other HPTLC-applications show a comparable range of linearity^34^,^35^,^36^. Besides the calibration also the accuracy of the values was investigated. LOD/LOQ were determined based on DIN standards (Table 2). The comparatively high relative standard deviation (RSD) is caused by the comparison between different single measurements, so analysis may differ between the batches but show good reliability inside the batch. As HPTLC is an open system, different exterior influences affect the measurements. This is why samples and standards of different concentrations must be analyzed in a single batch. Instead of regarding the RSD of LOD/LOQ, the coefficient of determination (R^2^) and its RSD would be a better indicator to prove the reliability of HPTLC-AS (Table 2). Additionally the determination of the recovery rate of lysozyme in white wine matrix was assigned (Supplementary Information Figure S3). Since the chromatographic analysis of samples containing phenolic compounds showed superior results on RP-18W plates, the determination of the recovery rate in the presence of white wine matrix was executed with RP-18W plates. Recoveries between 97 and 118% were determined. So, HPTLC-AS proved its feasibility also for a semi-quantitative estimation in presence of (food) matrix.

## Discussion

The use of aptamers in HPTLC analysis offers a broad range of advantages. While both, antibodies and aptamers, offer a high affinity and specificity towards various targets, the development of corresponding aptamers is also possible for toxic and non-immunogenic targets. Furthermore, the use of aptamers is also possible under non-physiological conditions and a wide range of modifications is commercially available, e.g., labelling with fluorophores or the integration of artificial nucleic acids.

The novel detection (HPTLC-AS) possesses various benefits, especially with regard to the *in vitro* production of aptamers. On the other hand, the affinity of the aptamers is strongly dependent on the conformation of the target compound: highly degraded samples such as denatured or proteolytic digested proteins cannot be detected using aptamers with specificity towards the intact protein. Here, the use of antibodies where antigens exhibit structural as well as sequential epitopes, still seems to be more advantageous.

The detection and semi-quantitative estimation of lysozyme in presence of a wine matrix is promising. A further development has to include an additional improvement of the sensitivity, e.g., by coupling of enzymes (horseradish peroxidase or alkaline phosphatase) to an aptamer or the implementation of DNAzymes into the sequence of the aptamer. The use of an indirect procedure using probes or intercalating dyes might be an innovative approach, as well. Further studies might also focus on the investigation of different wines and its content of fining agents using the presented HPTLC-AS methodology. In addition to the presented application of analyzing proteins, HPTLC-AS could also be, *vice versa*, an interesting tool for the characterization of aptamers in general. The methodology introduced was demonstrated for proteins, but should be, in principle, applicable for all kinds of analytical questions where thin-layer chromatography can be applied.

## Methods

### Aptamer Selection

#### Systematic Evolution of Ligands by Exponential Enrichment (SELEX)

The selection of aptamers with an affinity towards lysozyme (from hen’s egg-white) was performed by *Just-in-Time*-Selection in a buffer mimicking white wine conditions (1.5 mM potassium chloride, 14.5 mM potassium acetate, 3.5 mM magnesium acetate, 1.5 mM calcium acetate, 1 mM sodium dihydrogenphosphate, 4 mM potassium dihydrogenphosphate, 11.5% (v/v) ethanol, pH 3.1)^18^,^19^,^20^. Lysozyme was immobilized on magnetic particles so that following steps can be accomplished with a semi-automated magnetic separator (KingFisher Duo, Thermo Fisher Scientific Oy, Vantaa, Finland). The consecutive amplification was performed using BEAMing (beads, emulsion, amplification, magnetic) to enable an amplification with a lower number of by-products. Furthermore, the use of this technique combines amplification and strand separation in a single step^37^. After 15 turns, the resulting aptamers were sequenced and characterized regarding their affinity towards lysozyme by surface plasmon resonance (SPR). Based on the sequences obtained, fluorescently labelled aptamers were purchased from Sigma-Aldrich Chemie GmbH (Steinheim, Germany).

#### High-performance thin-layer chromatography (HPTLC)

HPTLC silica gel 60, silica gel 60 RP-18W F254s, RP-18, RP-8 F254s, and cellulose plates were obtained from Merck KGaA (Darmstadt, Germany).

#### Sample preparation

As model proteins lysozyme from hen’s egg-white, porcine pancreatic insulin, and bovine β-lactoglobulin were chosen. Because of their solubility, lysozyme and β-lactoglobulin were dissolved in double distilled water (ddH_2_O), whereas the aqueous solution of insulin contained 0.1% formic acid. Extraction of the commercial fining agent and the powdered egg-white was executed with ddH_2_O or the white wine-mimicking buffer, respectively. Commercially purchased white wine was spiked with 3 mg/mL lyosozyme-containing fining agent. After incubation on an orbital shaker (80 rpm, 30 min), samples were centrifuged (2200 × *g*, 5 min) and the supernatant was used for the chromatographic analysis. A commercially-available fining agent, containing high amounts of lysozyme, was a product of a European manufacturer (ViniPlus, Eaton Corp., Dublin, Ireland).

#### Chromatography

For the chromatographic separation, silica gel and cellulose as well as different reversed-phase (RP) materials were used. Silica gel HPTLC plates were pre-washed with methanol and activated at 100 °C for 10 min, while RP and cellulose HPTLC plates were used without pre-treatments. The samples were applied as bands (6 mm) using an HPTLC autosampler (ATS4, CAMAG AG, Muttenz, Switzerland). Separation was carried out in a twin-trough chamber with specific solvent mixtures depending on the stationary phase. Chromatography was consistently performed up to a migration distance of 70 mm. Finally, the remaining solvents were evaporated.

Analysis on silica gel HPTLC plates was performed using a solvent system consisting of a mixture of 2-butanol/pyridine/acetic acid/ddH_2_O (3.9/2/1/3.1; v/v/v/v). Cellulose plates were developed with a mixture consisting of 2-butanol/pyridine/ammonia/ddH_2_O (3.9/1/2/3.1; v/v/v/v). For the chromatographic separation on RP layers, a mixture containing acetonitrile/TFA/ddH_2_O (5/0.375/4.625; v/v/v) was applied. For determining the statistical parameters, (model) protein solutions were applied in different concentrations, but with the same sample volume.

#### Post-chromatographic derivatization with fluorescamine/ninhydrin

Usually, proteins are detected on TLC plates with non-specific dyes. Here, post-chromatographic derivatization was performed with fluorescamine (0.05% in acetone) or ninhydrin (0.5% in pyridine/acetic acid 5:1; v/v) staining solution. Ninhydrin was exclusively used for detection of proteins on cellulose plates. Subsequent to chromatography, the developed and dried plate was dipped in ninhydrin staining solution for 2 sec and evaporation was carried out at room temperature for 10 min. Afterwards, the plate was heated to 70 °C for 5 min. Proteins appear as violet bands on white background. For all other plates, fluorescamine was used as staining reagent. The dried plate was dipped in fluorescamine staining solution and evaporation was carried out at room temperature for 10 min. By using a TLC visualizer (CAMAG AG, Muttenz, Switzerland), proteins were detected at white light (ninhydrin) or ultraviolet light (UV 366 nm; fluorescamine), respectively.

#### Post-chromatographic derivatization using aptamers

To obtain a more specific, highly sensitive detection in comparison to the universal protein derivatization, a selective detection of single proteins was performed with the aptamer based staining protocol, the so called HPTLC-*aptastaining* (AS). The developed protocol was adapted according to traditional immunoblot procedures and applied for all stationary phase materials.

Following the evaporation of the mobile phase, the plate was transferred into a small dish. To inhibit unspecific bindings of the aptamers to the surface, the HPTLC plate was incubated with Tween^®^20 as blocking reagent, initially. Therefore, white wine buffer with 0.6% Tween^®^20 was applied and the plate was incubated two times for 5 min according to a modified western blot procedure presented in Steinhoff *et al*.^38^. Afterwards, the plate was incubated for 1 min with the aptamer solution containing fluorescently labelled aptamers (5 nM for silica gel and cellulose plates; 0.5 nM for RP phases), followed by a terminal washing step with white wine buffer for 30 sec. All incubation procedures were performed on an orbital shaker (Kombischüttler KL2, Edmund Bühler GmbH, Hechingen, Germany) at 70 rpm and room temperature.

Subsequently, the plate was dried in darkness, because of the coupled fluorophore’s light sensitivity. Documentation was performed using an imaging system (Versa Doc Gel Imager, Bio Rad Inc., Hercules, USA) at the specific wavelength of the fluorophore (550 nm for ATTO 550).

#### Electrophoresis

To compare the developed methodology with an established technique, protein samples were also analyzed with SDS-PAGE. For the electrophoretic separation, discontinuous-SDS-PAGE was performed using a modified protocol^39^. Gels with 14% polyacrylamide were used with 4% stacking gels. Sample extraction was performed as described above (sample preparation) and mixed with SDS sample buffer containing Tris-HCl (0.1 M), glycerol (20%), SDS (4%), 2-mercaptoethanol (4%), and bromophenol blue (0.5%) to a concentration of 0.5 mg/mL protein. Prior to application and separation, samples were incubated for 5 min at 95 °C. A commercially available protein standard (Precision Plus Protein^TM^ Standard, BioRad, Hercules, USA) was used. Electrophoresis was performed with 200 V, 70 mA and 300 W with duration of 55 min, and the proteins were visualized with Coomassie blue by following a staining protocol according to Candiano *et al*.^40^.

#### Statistical analysis

Validation of the TLC method was performed with regard to following parameters: linearity, limit of detection (LOD), limit of quantification (LOQ), and accuracy. Furthermore, the recovery rate was calculated in presence of matrix. Data processing was done with the software Image J (Open Source), subsequent to the documentation of the plate^41^. Linearity was verified by means of Mandel’s fitting test. It was validated using a nine-point calibration curve (each point with n = 5). Homogeneity of variance was assigned regarding all values obtained. The limit of detection (LOD), detection capability, and limit of quantification (LOQ) were determined using the calibration method. Accuracy was determined by analyzing the results of 15 individual measurements. Recovery in white wine matrix was calculated in triplicate at three different concentration levels. All remaining analyses were repeated at least twice to ensure reproducibility^42^,^43^,^44^.

## Additional Information

**How to cite this article**: Morschheuser, L. *et al*. HPTLC-*aptastaining* - Innovative protein detection system for high-performance thin-layer chromatography. *Sci. Rep.* **6**, 26665; doi: 10.1038/srep26665 (2016).

## Supplementary Material

Supplementary Information

## Figures and Tables

**Table 1:** Aptamer sequences with an affinity to lysozyme^20^.

**Aptamer**	**Sequence (5′-3′)**	**K**_**D**_ **[nM]**
LysApt4	CATCCGTCACACCTGCTCTTGTTATTTTTTGTTGATGTAGGTT	21.72
	TGATGATGTATTTCCGGTGTTCGGTCCCGTATC	
LysApt5	CATCCGTCACACCTGCTCCCTGCTAGAATTTTTCATGATCTTG	17.90
	CTGTATTTCTATTATGGTGTTCGGTCCCGTATC	

**Table 2:** Statistical parameters of HPTLC-AS on silica gel phases, using aptamer LysApt5.

**Synonym**	**Value**	**RSD**
Limit of detection	63.20 ng	18.64%
Limit of quantification	112.00 ng	19.35%
Accuracy	102.65%	5.43%
Coefficient of determination	0.991	0.6%
