# Supplementary material for: HPTLC-aptastaining – Innovative protein detection system for high-performance thin-layer chromatography
Source: Sci Rep. 2016 May 25;6:26665. doi: 10.1038/srep26665 (PMC4879557; doi:10.1038/srep26665)
Supplement: Supplementary Information [file srep26665-s1.pdf]

Supplementary Information

HPTLC-*aptastaining* – Innovative protein detection system for high-performance thin-layer chromatography

Lena Morschheuser[#], Hauke Wessels[#], Christina Pille, Judith Fischer, Tim Hünniger, Markus Fischer, Angelika Paschke-Kratzin, and Sascha Rohn[*]

University of Hamburg, Hamburg School of Food Science, Institute of Food Chemistry, Grindelallee 117, D-20146 Hamburg

[#] These authors contributed equally to this work.

* Corresponding author:

Prof. Dr. Sascha Rohn, Tel.: + 49 40 42838 7979, Fax: + 49 40 42838 4342, Email: rohn@chemie-uni-hamburg.de

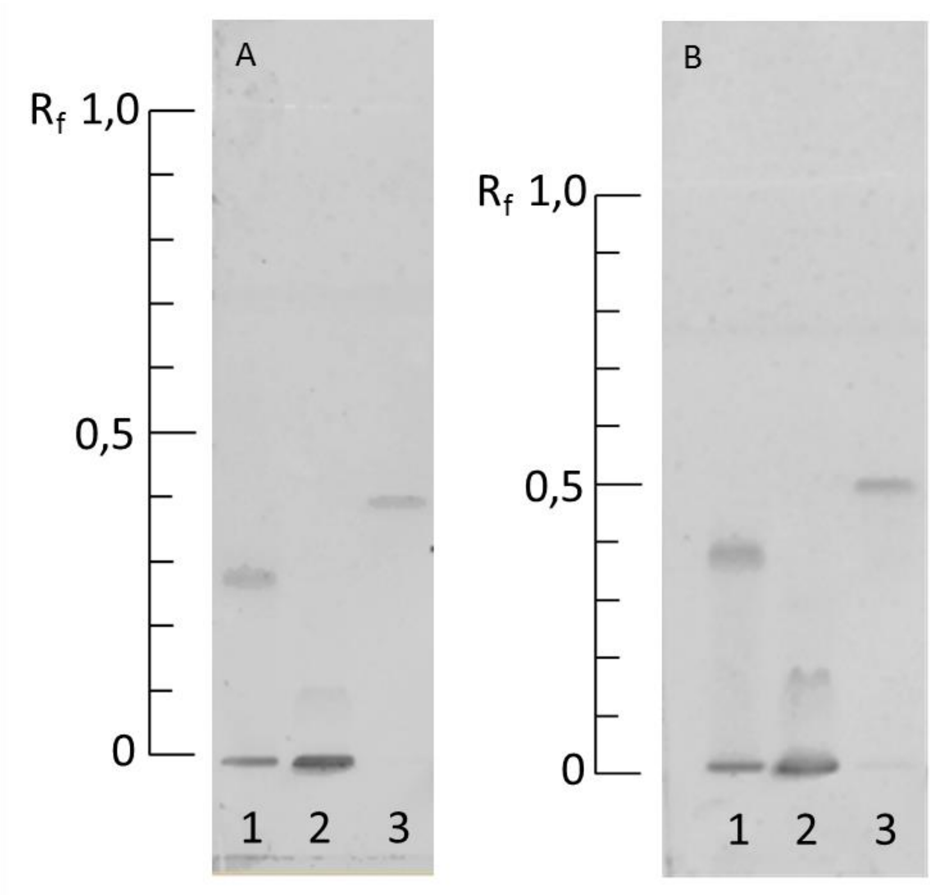

Figure S-1: HPTLC-*aptastaining* (HPTLC-AS) of model proteins on (A) RP-8 and (B) RP-18. *Aptastaining* was performed using the aptamer LysApt5 (λ=550 nm).
Legend: (1): lysozyme, (2): β-lactoglobulin, (3): insulin.

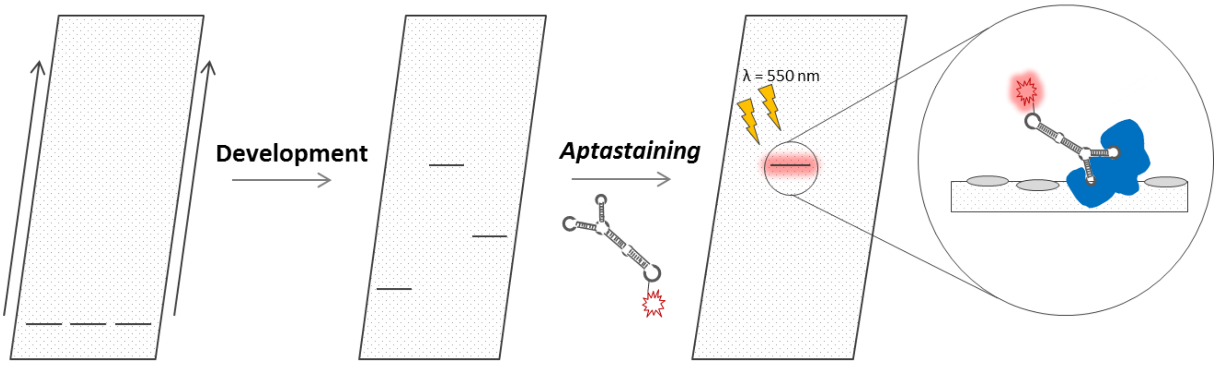

Figure S-2: Schematic overview of the developed *aptastaining* procedure using fluorescently labeled aptamers.

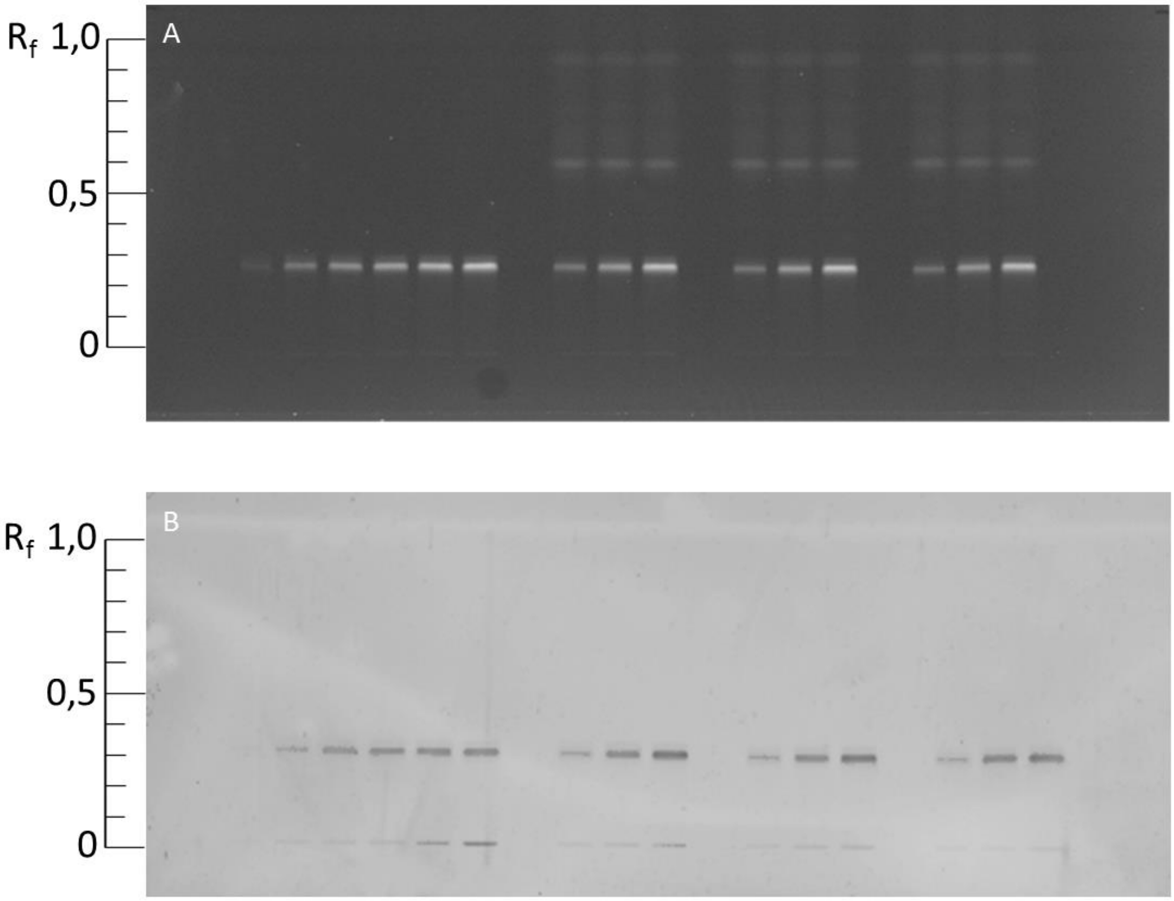

Figure S-3: HPTLC chromatogram of lysozyme (0.25-1.5 µg) dissolved in white wine for matrix calibration (RP-18W). (A) presents an unselectively stained HPTLC separation (fluorescamine staining solution, λ=366 nm). *Aptastaining* (B) was performed using the aptamer LysApt5 (λ=550 nm).
